# Effectiveness of Syrebo’s Glove Rehabilitation Program in a Patient With Middle Cerebral Artery Infarct: A Case Report

**DOI:** 10.7759/cureus.59314

**Published:** 2024-04-29

**Authors:** Vaishnavi S Sharma, H V Sharath, Anam R Sasun

**Affiliations:** 1 Department of Paediatric Physiotherapy, Center for Advanced Physiotherapy Education & Research (CAPER) Ravi Nair Physiotherapy College, Datta Meghe Institute of Higher Education and Research (DU) Sawangi Meghe, Wardha, IND; 2 Department of Neuro-Physiotherapy, Center for Advanced Physiotherapy Education & Research (CAPER) Ravi Nair Physiotherapy College, Datta Meghe Institute of Higher Education and Research (DU) Sawangi Meghe, Wardha, IND

**Keywords:** rehabilitation, physiotherapy, syrebo’s rehabilitation, soft robotic gloves, bilateral mca infract

## Abstract

In India, stroke is a significant health concern, with an estimated prevalence of around 1.54% in adults over 20 years old. The incidence of stroke in India varies regionally but is generally high due to factors like hypertension and lifestyle changes. Ischemic strokes comprise the majority, particularly in the middle cerebral artery (MCA) territory. MCA stroke presents with diverse symptoms such as weakness, speech difficulties, and vision problems, emphasizing the need for comprehensive rehabilitation. Physiotherapy plays a vital role in addressing these challenges, focusing on strength, coordination, mobility, and independence through tailored interventions. Additionally, soft robotic gloves, such as Syrebo's rehabilitation, offer promising advancements in neurorehabilitation by enhancing motor recovery and functional abilities, particularly in improving grip strength and hand functionality, thus improving outcomes for stroke patients. This case describes a 66-year-old female presenting with sudden left-sided weakness, slurred speech, and facial deviation indicative of bilateral MCA territory infarct. After admission requiring ventilation and medication, imaging confirmed the diagnosis. Following stabilization, she underwent neurophysiotherapy for rehabilitation. Neurological examination revealed deficits in muscle tone, reflexes, cranial nerve function, language, and swallowing. Outcome measures indicated progress in rehabilitation. The case underscores the significance of timely diagnosis and personalized rehabilitation in optimizing outcomes for MCA territory stroke patients.

## Introduction

In India, stroke is a significant health concern, with an estimated prevalence of around 1.54% in adults over 20 years old. The incidence of stroke in India varies regionally but is generally high due to factors like hypertension and lifestyle changes. Approximately 85% of these are ischemic strokes, and over half of all ischemic strokes occur in the middle cerebral artery (MCA) territory [1]. MCA stroke, or middle cerebral artery stroke, is a type of ischemic stroke that occurs when the blood flow to the brain is blocked by a clot in the middle cerebral artery [2]. The MCA is the most common artery involved in acute stroke. It branches directly from the internal carotid artery and consists of four main branches, M1, M2, M3, and M4. These vessels provide blood supply to parts of the frontal, temporal, and parietal lobes of the brain, as well as deeper structures, including the caudate, internal capsule, and thalamus [3]. Its vast supply means that strokes involving the MCA territory can have a multitude of presenting symptoms, depending on which branches and structures are affected [4]. This type of stroke is the most common and can cause a range of symptoms, including weakness or paralysis on one side of the body, difficulty speaking or understanding speech, and vision problems [5].

There are multiple risk factors for strokes, which can be divided into modifiable and non-modifiable categories - many of the causes of hemorrhagic and ischemic stroke overlap. The non-modifiable risk factors of each include age, sex, race, and genetics. The risk increases as you get older; the risk is higher in men at a younger age, but the risk of death is higher overall in women [6]. The effect of physiotherapy rehabilitation in MCA stroke is a crucial aspect of patient care, aiming to address the diverse physical and functional challenges that arise from this type of stroke [7]. Physiotherapy interventions are designed to help individuals regain strength, coordination, mobility, and independence following an MCA stroke. These interventions may include tailored exercise programs, gait training, balance and coordination exercises, and activities to improve motor control. It plays a key role in preventing complications, minimizing impairments, and maximizing functional recovery through early assessment and the employment of evidence-based interventions [8].

Soft robotic gloves, such as those used in Syrebo's rehabilitation, have shown promising results in augmenting motor recovery and functional abilities among stroke patients. Their integration with conventional rehabilitation approaches enhances the effectiveness of neurorehabilitation by improving grip strength and fostering greater functional independence. This underscores the significance of incorporating such innovative technologies alongside established rehabilitation methods to optimize patient outcomes [9]. These gloves are designed to assist in rehabilitation by utilizing pneumatic pressure to flex and extend the human hand, allowing for the performance of repetitive exercises aimed at improving motor control and hand functionality. The lightweight and compact nature of soft robotic gloves makes them suitable for daily living activities and rehabilitation purposes [10].

## Case presentation

Patient information

We report a case of a 66-year-old female who presented to our casualty unit with complaints of sudden onset left-sided weakness, slurring of speech and deviation of angle of mouth to left side for the past eight hours. The other associated complaints include the inability to perform bed mobility activities and the inability to perform activities of daily living. The weakness was sudden in onset and gradually progressive in nature. Medical history revealed history of hypertension for the past three years along with dilated cardiomyopathy. The patient was intubated and placed on synchronized intermittent mandatory ventilation mode (SIMV) at the time of admission due to gradual reduction in oxygen saturation. 

Following this, patient was shifted to the Intensive Care Unit followed by application of Ryle’s tube and Foley’s catheter and medications like injection piperacillin/tazobactam, injection mannitol, injection optineuron, injection ecosporin, injection Rosuvas. Investigations like magnetic resonance imaging (MRI) revealed sharply defined wedge shaped intra axial hypodensity involving grey and white matter of the right fronto-parieto-temporal lobe suggestive of acute infarct corresponding to bilateral MCA territory along-with midline shift of 11 mm towards. After two days patient maintained her oxygen saturation levels and was on 6 litres of O2. Gradually she started maintaing her oxygen saturation at room temperature (Day 4). Following this, patient was referred to neurophysiotherapy for further rehabilitation. Informed consent was taken from the patient. A neurological examination was performed on Day 2.

On observation

**Figure 1:**
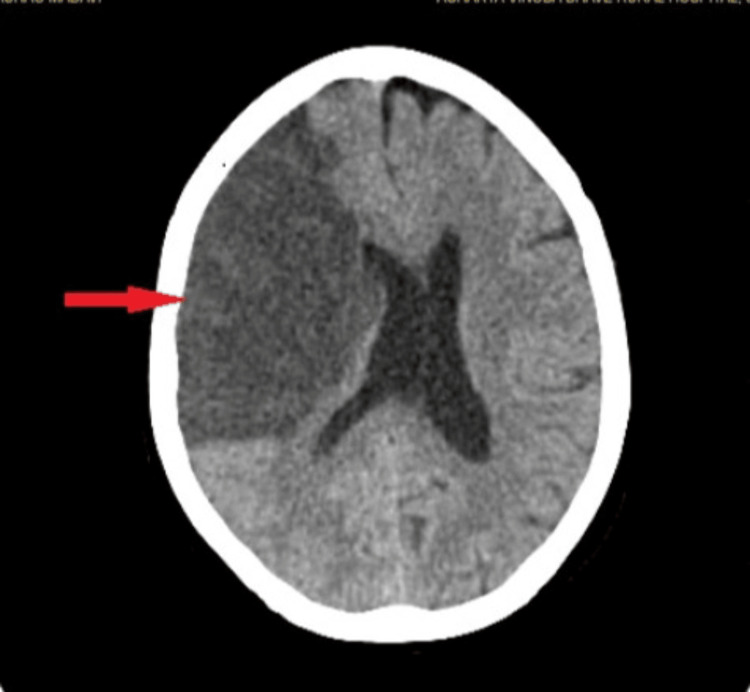
Right fronto-parieto-temporal lobe suggestive of acute infarct corresponding to bilateral middle cerebral artery (MCA) territory along with midline shift of 11 mm towards left side.

Before assessing patient, consent was taken. Facial asymmetry was observed with a deviation of the angle of the mouth to the left side. A mini mental state examination revealed patient was unconscious and not oriented. Muscle tone was decreased on the left side (hypotonia). All superficial, deep, and combined cortical reflexes were diminished (+1) and the plantar response was flexor. Cranial nerve examination revealed the involvement of the facial nerve (sensory and motor part), balance part of the vestibulocochlear nerve, Broca’s aphasia, and dysphagia. MRI report shows bilateral MCA in Figure 1.

Physiotherapy intervention

Therapeutic management is given in Table 1.

**Table 1:** Physiotherapy rehabilitation lasted for six weeks

Goals	Interventions
Inpatient Rehabilitation: (1 to 2 weeks)	
Education to patient’s relatives about the pathology	Educate the patient regarding the present health condition of the patient and the physiotherapy protocol to be implemented. The patient should be helped to develop coping skills to deal with the current quality of life. Coping skills include stress management techniques and self-care practices.
Maintain awareness of weakened side	All forms of stimulus were done through the left side.
To facilitate a level of arousal	Multimodal Auditory Stimulation (30 minutes)
To avoid mal-alignments of anatomical structures	Effective positioning of hemiparetic posture, typically assumed (change position after every 2 hours)
To normalize tone of muscles (Lt)	Proprioceptive neuromuscular facilitation techniques (PNF) (D1-D2) pattern, Rhythmic initiation. (10 reps, 2 sets) Joint approximation techniques (10 reps, 2 sets) Weight-bearing exercises (bridging, prone on elbows were initiated).
To promote airway clearance	Chest PNF techniques and end-expiratory pressure were applied (10 reps, 2 sets)
To encourage bed-mobility	Bed mobility exercises specifically Log rolling were taught in the initial phase followed by transition activities (supine to side lying position)
Outpatient Rehabilitation (3 to 6 weeks )	
To improve gross motor task	Syrebo Rehabilitation gloves (30 repetitions, 3 sets)
Oropharyngeal retraining	Stroking over the neck area, ice massaging of the tongue, and stimulating lip closures were used to facilitate. Each exercise (10 reps, 2 sets)
To promote symmetry of the face	Facial exercises are mirror visual feedback exercises,facial muscle strengthening exercises, speech and articulation exercises, smile stretch, cheek puffing, straw exercise (10 reps, 2 sets)
To promote functional independence	Sit to Stand training (10 reps, 2 sets)
To improve movement control	Scapular PNF patterns (10 reps, 2 sets)
To improve static and dynamic trunk balance	Trunk and Pelvis PNF exercises( 10 reps, 2 sets)

**Figure 2:**
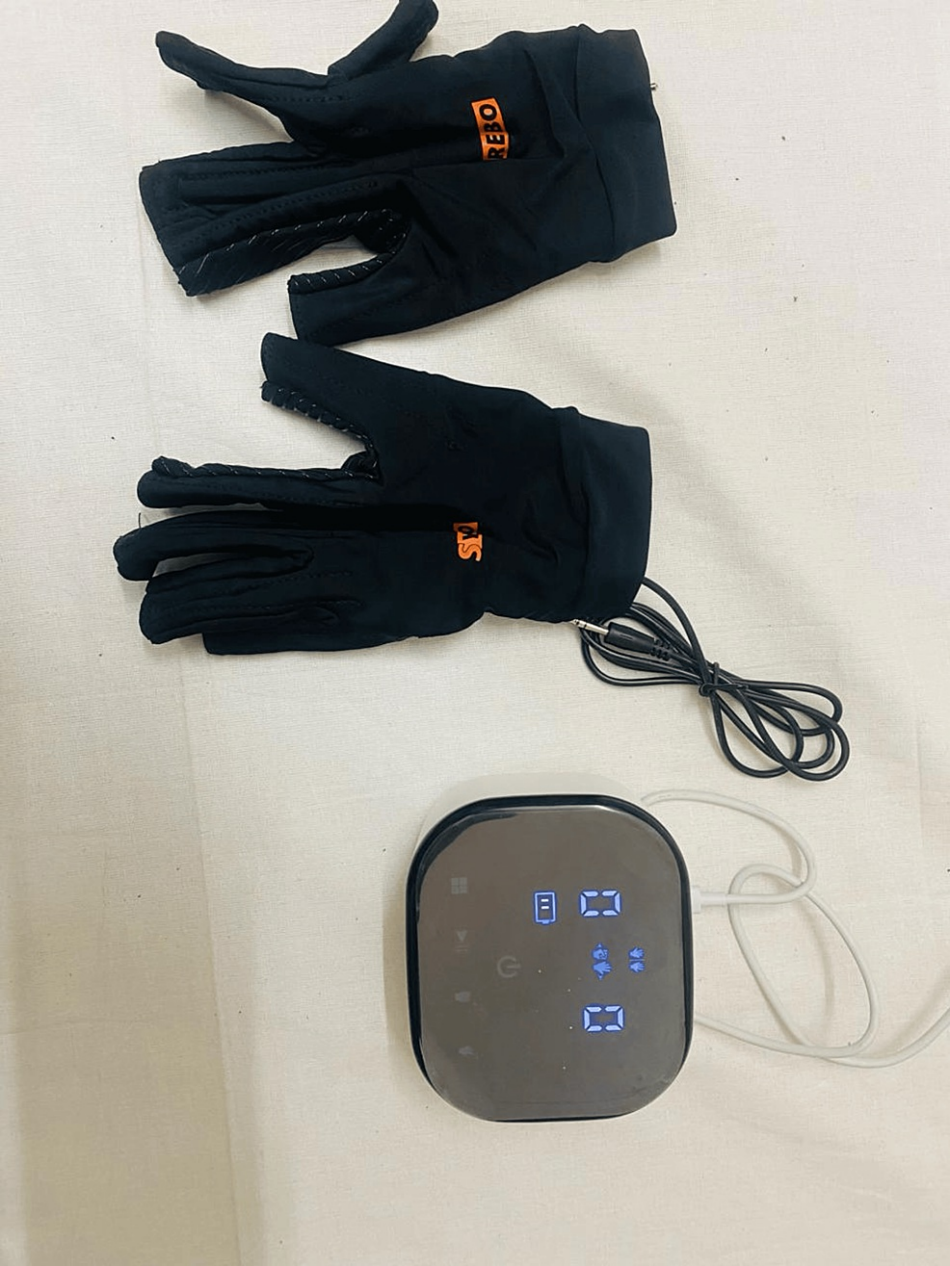
Soft robotic gloves

**Figure 3:**
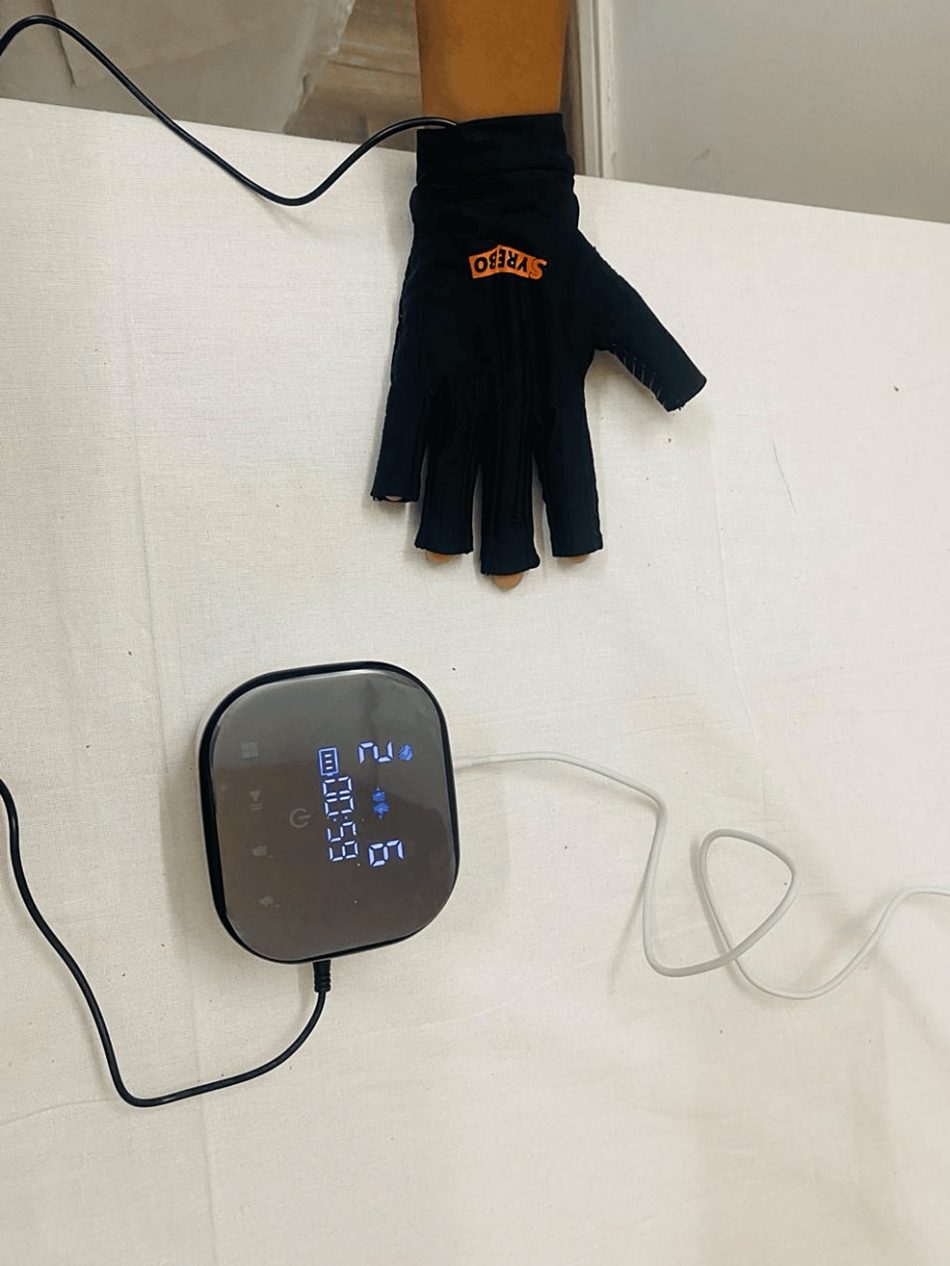
Patient using soft robotic gloves (Syrebo rehabilitation)

Figure 2 shows Syrebo's soft robotics glove equipment. Figure 3 shows the application of Syrebo's soft robotics gloves in patient hand. 

Follow-up and outcome measures

Outcome measures like the Glasgow Coma Scale (GCS), Tone Grading Scale, Functional Independence Scale, ICU Mobility Score and Brunnstrom Stages were used to assess pre- and post-rehabilitation and follow-up shown in Table 2.

**Table 2:** Follow-up and outcome measures

Sr.no	Outcomes measure	Pre-physical therapy rehabilitation (/N)	Post-physical therapy rehabilitation (/N)
1.	Glasgow coma scale	11/15	13/15
2.	Tone grading scale	1+	2+
3.	Functional independence scale	1/7	6/7
4.	ICU mobility score	1/10	7/10
5.	Brunnstrom stages	1/7	3/7

## Discussion

Middle cerebral artery infarct (MCAI) often leads to significant motor impairment, particularly in the upper extremities, affecting the patient's quality of life. Traditional rehabilitation methods have shown limited success in restoring function fully. Syrebo's glove rehabilitation program offers a promising alternative approach [11-14]. This case study aims to evaluate the effectiveness of this program in MCAI patients, focusing on motor recovery and functional improvement.

Motor recovery was assessed using standardized scales such as Brunnstrom stages. Significant improvements were observed in both upper extremity motor function and coordination post-intervention. The patients exhibited enhanced dexterity, grip strength, and range of motion in affected limbs, indicating the effectiveness of Syrebo's glove rehabilitation program in promoting motor recovery [15]. Spasticity is a common complication following MCAI, contributing to motor impairment. The program incorporated techniques aimed at reducing spasticity, including proprioceptive neuromuscular facilitation (PNF) and stretching exercises. Post-intervention assessments revealed a noticeable reduction in spasticity levels among the participants, leading to improved muscle tone and voluntary movement control [16-18].

Activities of daily living (ADL) performance is crucial for the independence and quality of life of stroke survivors. Following Syrebo's glove rehabilitation program, patients demonstrated enhanced performance in various daily tasks such as dressing, feeding, and grooming. The program's focus on task-specific training facilitated the transfer of motor skills into functional abilities, empowering patients to engage more actively in their daily routines [19]. The sustainability of rehabilitation outcomes is a critical consideration in stroke management. Follow-up evaluations conducted at intervals beyond the intervention period demonstrated the maintenance of gains achieved through Syrebo's glove rehabilitation program. Continued engagement in home-based exercises and periodic reinforcement sessions contributed to sustaining motor recovery and functional improvements over time [20].

Despite the overall positive outcomes, several challenges were encountered during the implementation of Syrebo's glove rehabilitation program. These included patient compliance, availability of resources, and variability in individual response to the intervention. Addressing these challenges necessitates personalized care plans and ongoing support from healthcare professionals to optimize outcomes for MCAI patients. This case study has certain limitations, including a small sample size and the absence of a control group for comparison. Future research endeavors should aim to conduct larger-scale clinical trials with randomized controlled designs to validate the efficacy of Syrebo's glove rehabilitation program across diverse patient populations. Additionally, exploring the program's integration with other rehabilitation modalities and its long-term impact on functional independence warrants.

## Conclusions

Syrebo's glove rehabilitation program emerges as a promising intervention for promoting motor recovery and functional improvement in MCAI patients. Through a combination of targeted exercises and task-specific training, the program addresses the complex rehabilitation needs of stroke survivors, empowering them to regain independence and enhance their quality of life. Further research and clinical validation are necessary to establish its widespread adoption and integration into standard stroke rehabilitation protocols.
